# Ascorbic Acid Attenuates Senescence of Human Osteoarthritic Osteoblasts

**DOI:** 10.3390/ijms18122517

**Published:** 2017-11-24

**Authors:** Maximilian G. Burger, Amir Steinitz, Jeroen Geurts, Benjamin E. Pippenger, Dirk J. Schaefer, Ivan Martin, Andrea Barbero, Karoliina Pelttari

**Affiliations:** 1 Department of Biomedicine, University of Basel, University Hospital of Basel, 4031 Basel, Switzerland; maximilian.burger@usb.ch (M.G.B.); amir.steinitz@usb.ch (A.S.); ivan.martin@usb.ch (I.M.); karoliina.pelttari@usb.ch (K.P.); 2 Department of Plastic, Reconstructive, Aesthetic and Hand Surgery, University Hospital of Basel, University of Basel, 4031 Basel, Switzerland; dirk.schaefer@usb.ch; 3 Departments for Orthopedic Surgery and Traumatology, University Hospital of Basel, 4031 Basel, Switzerland; 4 Departments Spine Surgery and Biomedical Engineering, University Hospital of Basel, University of Basel, 4031 Basel, Switzerland; jeroen.geurts@usb.ch (J.G.); benjamin.pippenger@straumann.com (B.E.P.)

**Keywords:** osteoblast, osteoarthritis, oxidative stress, senescence, subchondral bone, transcriptomics

## Abstract

The accumulation of senescent cells is implicated in the pathology of several age-related diseases. While the clearance of senescent cells has been suggested as a therapeutic target for patients with osteoarthritis (OA), cellular senescence of bone-resident osteoblasts (OB) remains poorly explored. Since oxidative stress is a well-known inducer of cellular senescence, we here investigated the effect of antioxidant supplementation on the isolation efficiency, expansion, differentiation potential, and transcriptomic profile of OB from osteoarthritic subchondral bone. Bone chips were harvested from sclerotic and non-sclerotic regions of the subchondral bone of human OA joints. The application of 0.1 mM ascorbic acid-2-phosphate (AA) significantly increased the number of outgrowing cells and their proliferation capacity. This enhanced proliferative capacity showed a negative correlation with the amount of senescent cells and was accompanied by decreased expression of reactive oxygen species (ROS) in cultured OB. Expanded cells continued to express differentiated OB markers independently of AA supplementation and demonstrated no changes in their capacity to osteogenically differentiate. Transcriptomic analyses revealed that apoptotic, cell cycle–proliferation, and catabolic pathways were the main pathways affected in the presence of AA during OB expansion. Supplementation with AA can thus help to expand subchondral bone OB in vitro while maintaining their special cellular characteristics. The clearance of such senescent OB could be envisioned as a potential therapeutic target for the treatment of OA.

## 1. Introduction

Osteoarthritis (OA) is the most prevalent degenerative joint disorder and a main reason for chronic pain and disability in the elderly population [1]. While multiple factors, such as genetic disposition, mechanical stress (caused by overload, obesity, or joint malalignment), previous injuries, or age are known to increase the risk to develop OA [2], the precise etiology of this multifactorial disorder remains elusive. Characteristically, OA is defined by progressive cartilage degeneration [3], alterations of the cartilage–bone interface [4], inflammation of the synovial tissue [5], formation of osteophytes, and bone sclerosis [6].

Recently, the cartilage underlying subchondral bone has been ascribed a crucial role in the progression of OA [7,8]. While at earlier phases of OA subchondral bone volume is decreased [9], its substantial thickening, the so-called sclerosis, characterizes later stages of OA [6]. The temporal sequence of bone and cartilage changes might vary according to the disease phenotype or the animal model, however it is commonly agreed that the interplay between these tissues is crucially involved in the development of OA [10,11]. In this regard, in vitro studies have demonstrated that osteoblasts (OB) from patients with OA significantly influenced the metabolic activity of human chondrocytes [12,13], e.g., by upregulating the expression of extracellular matrix degrading enzymes, such as matrix metalloproteinase-3 (MMP-3) and matrix metalloproteinase-13 (MMP-13) [14,15]. This can be most possibly due to the altered characteristics of OB from osteoarthritic tissues, e.g., in terms of their abnormal production of extracellular matrix components [16] or of other factors, such as cartilage degrading mediators and growth factors, as compared to OB from healthy subchondral bone [17,18,19].

Cellular aging is defined as the progressive decline in the resistance of cells to stress, characterised by distinct hallmarks, such as the aggregation of reactive oxygen species (ROS), the loss of protein homeostasis, mitochondrial dysfunction, DNA damage, and epigenetic alterations [20]. The accumulation of these events will ultimately result in senescent cells, which cease proliferation and start expressing pro-inflammatory and extracellular matrix-degrading factors [21]. Senescent cells, typically occurring during natural aging, have been found to highly accumulate in musculoskeletal diseases, including OA and osteoporosis [22,23]. While replicative senescence has been described in osteoblasts of OA patients in vitro [24], a biological role of senescence on bone formation was addressed only very recently, demonstrating that the elimination of senescent cells in mice prevented not only age-related disorders in general [25], but also bone loss [22]. However, the contribution of specific senescent cells, such as OB, to this phenomenon remains unclear because of their systemic clearance. For chondrocytes, which are another cell type present in the synovial joint, the role of senescence and its possible function on age-associated modifications of cartilage, and thus on the development of osteoarthritis, has been addressed in some more studies [26,27,28]. In particular, such premature *chondrosenescence* is proposed to contribute to a pro-inflammatory and catabolic environment [29], altering intercellular communication and compromising the surrounding extracellular matrix thus promoting the onset of OA [30,31]. In line with these findings, a recent study impressively demonstrated that the local clearance of senescent cells in vivo attenuated the development of post-traumatic osteoarthritis in mice [32]. 

Free radicals and the associated oxidative stress are known as major inducers of cellular senescence [33] and also seem to play a major role in the process of impaired functionality and decreased number of osteoblasts during aging, as shown in vitro and in vivo in numerous studies [34,35,36]. Moreover, it has been highlighted that a decrease in new bone formation and osteoblastic activity is caused by increased levels of free radicals and is associated in vitro with increased ROS levels leading to the activation of p53 and p66^shc^, key components influencing apoptosis pathways and lifespan [37].

Since the production of free radicals such as ROS has been proposed to cause senescence, counteracting oxidative stress could represent a possible mean to increase the life span of OB. Accordingly, it has been demonstrated that the supplementation of antioxidants such as ascorbic acid could have a beneficial effect on the reproduction and differentiation of osteoblastic cell lines, enhancing mesenchymal stem cell proliferation and maintaining their characteristic phenotype in vitro [38,39]. However, it remains unaddressed whether the supplementation of antioxidants can reduce or delay the number of senescent cells in primary OB derived from OA patients, while maintaining their phenotypical characteristics.

The aim of our study was to investigate the effect of the antioxidant Ascorbic acid-2-phosphate (AA) supplementation on the isolation efficiency, expansion, and differentiation potential of human OB from osteoarthritic subchondral bone. We furthermore examined the onset of senescence in the presence and absence of AA and studied the entailed modulation of the transcriptomic profiles of primary osteoarthritic OB.

## 2. Results

### 2.1. Effect of Ascorbic Acid on the Outgrowth and Proliferation Rate of Human Osteoarthritic OB

**Figure 1:**
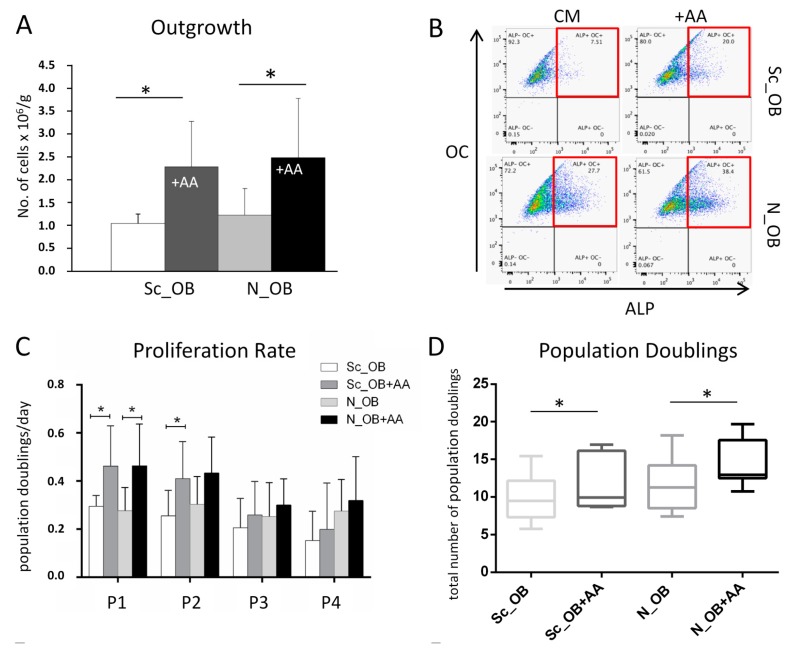

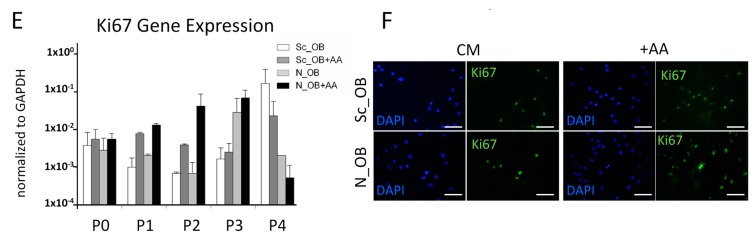
Effect of ascorbic acid-2-phosphate (AA) on the outgrowth and proliferation of osteoarthritic osteoblasts (OB). (**A**) The number of outgrowing cells from subchondral bone chips from sclerotic (Sc_OB) and non-sclerotic regions (N_OB) was quantified and normalised per gram of bone tissue in standard culture medium and in the presence of AA (+AA; for *n* = 5–7 donors/experimental group, analysed in triplicates for each donor); (**B**) Expression profile determined by FACS of alkaline phosphatase (ALP) and osteocalcin (OC) in isolated Sc_OB and N_OB, following outgrowth (P0) in the absence (CM) or presence of AA (+AA); (**C**) Proliferation rate, defined by the number of population doublings (PD)/day, of expanded Sc_OB and N_OB (*n* = 7 donors, analysed in duplicates for each donor); (**D**) Total number of population doublings before ceasing proliferation by the end of passage 4; (**E**) Expression of the proliferation marker Ki67 at the mRNA level (normalised to the housekeeping gene GAPDH) and (**F**) at the protein level in P2 expanded cells, determined by immunofluorescence. Scale bar = 100 µm. Data in (**A**,**C**–**D**) are presented as mean ± SD (standard deviation). Sc_OB = sclerotic osteoblasts; N_OB = non-sclerotic osteoblasts; CM = culture medium; +AA = culture medium supplemented with 0.1 mM ascorbic acid. * indicates statistically significant differences (*p* < 0.05).

First, we assessed the influence of AA on the outgrowth efficiency of human primary OB harvested from sclerotic (Sc_OB) and non-sclerotic (N_OB) osteoarthritic subchondral bone. A significantly increased number of OB (per gram of tissue) was observed to grow out from bone chips in the presence of AA (2.5 ± 1.1-fold increase, *p* = 0.0072 for Sc_OB, and 2.1 ± 0.6-fold increase; *p* = 0.0135 for N_OB) as compared to the number of OB growing out from the corresponding bone chips cultured in standard culture medium (CM) (Figure 1A). Conversely, statistically significant differences were not detected between the two OB types regarding the mean number of outgrowing cells either at control conditions (CM) or in the presence of 0.1 mM AA (Figure 1A). 

The expression of the osteogenic cell markers alkaline phosphatase (ALP) and osteocalcin (OC) was confirmed in standard culture conditions by fluorescence-activated cell sorting (FACS) analysis for the outgrowth of Sc_OB (7.5% of the CD45 negative population) and N_OB (27.7% of the CD45 negative population). In the presence of AA (+AA), slightly larger percentages of CD45^−^/ALP^+^/OC^+^ cells could be identified for Sc_OB (20.0%) and N_OB (38.4%) (Figure 1B).

At the gene expression level, significant differences were not observed between Sc_OB and N_OB in the presence or absence of AA, for OC, Col1A1 and 1A2, or TGFβ1 by quantitative RT-PCR. Irrespective of their sclerotic or non-sclerotic origin, OB demonstrated a significantly increased capacity to proliferate in the presence of AA (1.6 ± 0.6 and 1.9 ± 0.9-fold increase for Sc_OB at P1 (*p* = 0.0313) and P2 (*p* = 0.0469), respectively; 1.7 ± 0.5-fold increase for N_OB at P2, *p* = 0.0156) (Figure 1C). This trend of enhanced proliferation rate was maintained in the following passages when AA was supplemented to the culture medium, finally resulting in a significantly increased total number of accumulated population doublings by P4 (10.03 ± 3.2 PD for Sc_OB vs 12.38 ± 3.8 for Sc_OB+AA, *p* = 0.0156; 11.8 ± 3.6 PD for N_OB vs 14.3 ± 3.2 PD for N_OB+AA, *p* = 0.0313) (Figure 1D). This effect positively correlated with the increased expression of a master regulator of proliferation, Ki67, both at the gene expression level (up to 60-fold) (Figure 1E) and at the protein expression level (Figure 1F).

### 2.2. Attenuation of Human Osteoarthritic OB Senescence in the Presence of Ascorbic Acid

**Figure 2:**
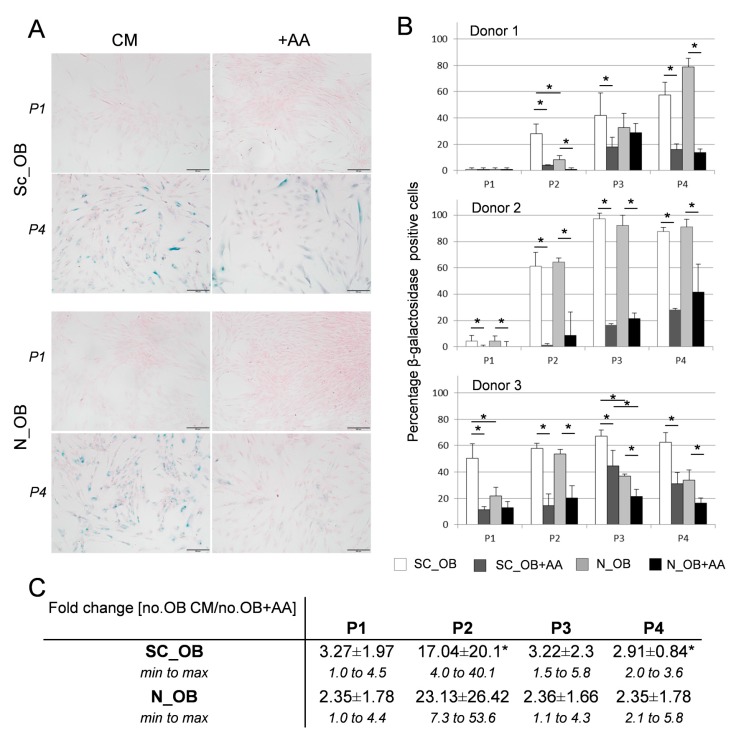
Effect of AA on cell senescence. (**A**) Senescent Sc_OB and N_OB were stained blue by senescence specific β-galactosidase staining at P1 and P4 (representative pictures shown for donor 1). Cells were counterstained with Nuclear Fast Red; scale bar = 200 µm (**B**) Quantification of positively stained cells versus the total amount of osteoarthritic OB in percentage, for three individual donors (donors 1–3, values are means (±SD) of four observed fields per experimental group); * indicates statistically significant differences (*p* < 0.05) between the displayed conditions and cell sources at each passage. (**C**) Indication of the x-fold higher expression (mean ± SD) of senescent cells in CM as compared to the same cells expanded with AA (percentage of senescent cells in CM/percentage of senescent cells in the presence of AA), with minimal and maximal fold-changes indicated; * indicates statistically significant differences (*p* < 0.05) between culture conditions for each cell type, *n* = 3 donors.

To evaluate a possible correlation between the demonstrated enhanced proliferation capacity of osteoarthritic OB and the amount of senescent cells occurring in the presence of AA, OB were stained at each passage in the course of expansion for senescence-specific β-galactosidase (shown representatively for P1 and P4 in Figure 2A). The number of positively stained cells was quantified for each condition and time point, and was presented individually for each donor as the percentage of senescent cells in the total population (Figure 2B). The application of AA significantly decreased the number of senescent cells. While at single-donor level some significant differences between Sc_OB and N_OB were determined when the OB were cultured in CM (for donor 1 at P2 and for donor 3 at P1 and P3), no such differences were found at any culture condition once the data from these three donors were analysed together. In fact, the number of senescent cells at each passage was always at least 2.4-fold higher for Sc_OB as well as for N_OB, when no AA was supplemented, while the strongest effect of AA was observed at P2 (17.04 ± 20.1 for Sc_OB, *p* = 0.0424) (Figure 2C).

Taken together, Sc_OB and N_OB responded in a similar way to the application of AA by diminishing the number of senescent cells.

**Figure 3:**
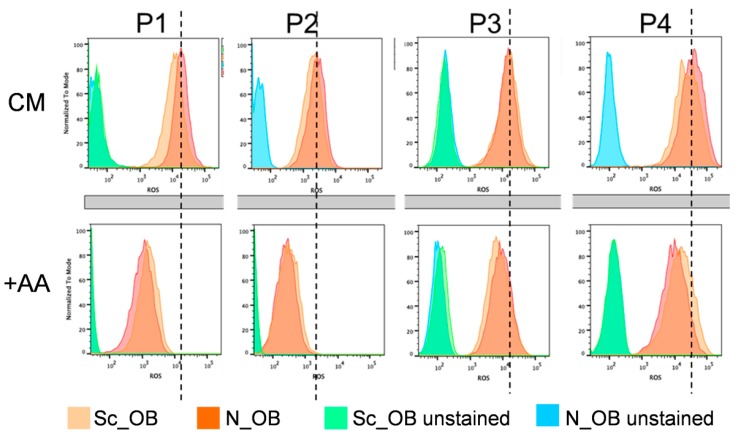
Effect of AA on reactive oxygen species (ROS) expression. The amount of ROS was determined by FACS for Sc_OB and N_OB, expanded in the absence (CM, upper panel) or presence of 0.1 mM AA (lower panel). The dashed lines visually depict the peak of ROS for Sc_OB (representative also for N_OB) in standard culture medium (CM).

We then investigated whether the attenuation of OB senescence in the presence of AA was mediated by the antioxidative function of AA, by quantification of intracellular reactive oxygen species (ROS) production. ROS expression was significantly decreased in the presence of AA by 13-fold ± 7.6 at P1, as compared to CM-expanded OB, and the extent of this reduction successively decreased with each passage from 8.9 ± 3.0 at P2, to 1.9 ± 0.4 at P3, to finally 2.7 ± 1.5 at P4 (Figure 3). Conversely, no significant differences in the mean fluorescence intensity, which was considered to directly correlate with the amount of ROS expression, were observed between Sc_OB and N_OB in the same culture conditions (either with or without AA).

Here, we demonstrated that the enhanced proliferative capacity of osteoarthritic OB in the presence of AA was accompanied by a reduced amount of senescent cells and a decreased expression of ROS.

### 2.3. Osteogenic Differentiation of Human Osteoarthritic OB in the Presence of Ascorbic Acid 

Osteoarthritic OB expanded for two passages in either the absence or presence of AA were cultured in standard 2D osteogenic medium for 21 days (*n* = 5 donors). Under control conditions, lacking the osteogenic factors dexamethasone and β-glycerophosphate, no mineralisation was detectable by alizarin red staining. Following osteogenic induction, variable degrees of mineralisation were observed for both Sc_OB and N_OB, ranging between no and strong mineralisation. The observed intradonor specific osteogenic differentiation ability of OB remained unchanged in the presence of AA.

**Figure 4:**
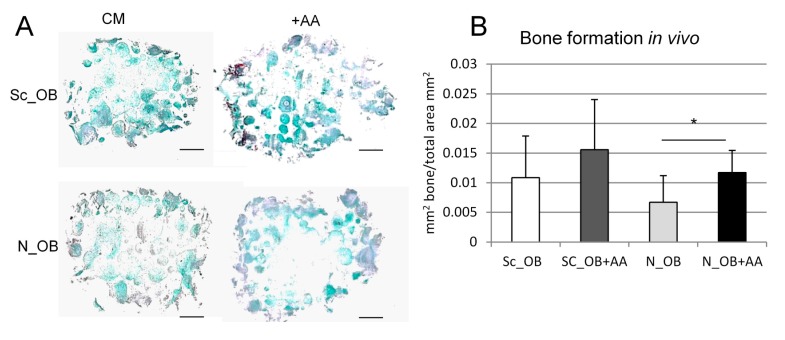
Effect of AA on OB differentiation. (**A**) Representative overview pictures of Masson trichrome-stained tissue of OB-seeded ceramic scaffolds (Engipore) implanted subcutaneously, taken 4 weeks after implantation; Scale bar = 1 mm; (**B**) Quantification of the corresponding bone-like areas, expressed as the ratio to the total area (mm^2^/mm^2^) (mean ± SD of eight sections per experimental group); * indicates a statistically significant difference (*p* < 0.05).

To investigate the bone formation potential of osteoarthritic OB in vivo, cells seeded on a ceramic scaffold (Engipore) were implanted in subcutaneous pouches of nude mice. Masson trichrome staining of explanted construct 4 weeks postimplantation did not reveal mature bone formation, however areas of pre-bone were identified (Figure 4A) and quantified by bone histomorphometry (Figure 4B). In general, a similar bone formation capacity was identified for all OB types expanded under different conditions, although significantly more bone-like tissue was found in scaffolds seeded with N_OB+AA as compared to N_OB.

These results indicate that supplementation of OB with AA during outgrowth and expansion did not result in reproducible changes of their osteogenic differentiation capacity.

### 2.4. Transcriptomic Analysis of Human Osteoarthritic OB in the Absence and Presence of Ascorbic Acid

To assess the effect of AA supplementation on osteoarthritic OB at molecular levels, RNAseq-based transcriptomic profiling was performed following outgrowth (P0) or expansion (at P2 and P4). For this analysis, Sc_OB and N_OB were not discriminated since no major differences were observed between these two osteoarthritic OB populations in the experiments described before.

**Figure 5:**
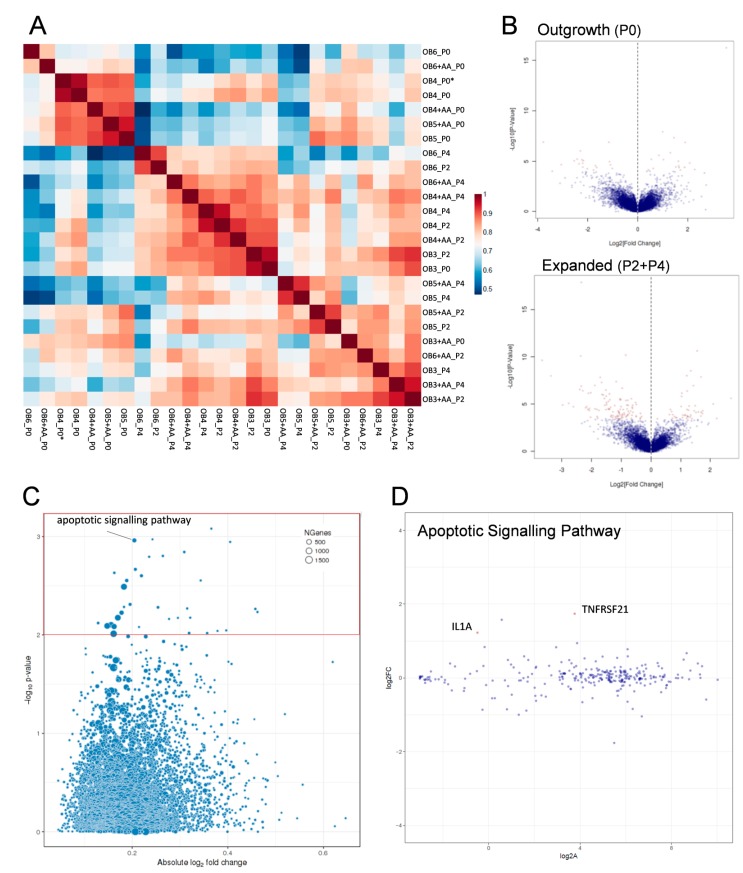
Transcriptomic analysis. The effect of AA on the mRNA expression profiles of osteoarthritic OB was assessed by RNAseq in the absence and presence (+AA) of AA following outgrowth (P0) and expansion (P2, P4) for *n* = 4 donors. (**A**) Gene expression heat maps for each donor (donors OB3, OB4, OB5, and OB6) at different time points in the absence or presence of AA (+AA) are depicted with the color scheme shown on the right; * indicates a replicate sample from the same donor (donor 4, OB4_P0). (**B**) Volcano plot visualising the differentially expressed genes in outgrowing OB (corresponding to P0 cells, upper plot) and expanded OB (including P2 and P4 OB, lower plot). Red dots depict significantly differentially expressed genes between OB cultured with AA as compared to OB in CM. (**C**) The main pathways affected by AA supplementation in expanded OB, as identified by GO GeneSet downstream analyses, are shown in this scatter plot, with the most affected ones included within the red box (*p* < 0.01). (**D**) Gene expression of members of a selected pathway, the apoptotic signalling pathway (indicated in **C**), showing the distribution of genes regulated in the presence of AA.

For three of the four donors (donors 4, 5, and 6), outgrowing OB (P0) were found to group according to their overall expression profile, whereas no such correlation was identified for P2- and P4-expanded OB (Figure 5A). Therefore, for further downstream analysis, P2- and P4-expanded OB were not discriminated, but analysed as one group representing the phenotype of expanded cells in the absence or presence (+AA) of AA. For outgrowing OB cultured in the presence of AA, 24 genes (14 genes downregulated and 10 genes upregulated; Supplemental Table S1) were identified to be differentially expressed as compared to CM-cultured cells (Figure 5B upper plot). In control conditions, among the higher expressed transcripts were PTX3 (pentraxin 3), IGFBP1 (insulin-like growth factor-binding protein 1), and SESN2 (sestrin 2), whereas transcript levels of HAS1 (hyaluronan synthetase 1) and SEMA7A (semaphorin 7A) were decreased. Following expansion, significant differences were found for 44 genes (33 genes downregulated, 11 genes upregulated; Supplemental Table S2) between CM- and AA-expanded OB. Higher levels of transcripts were detected at control conditions, e.g., for LGR5 (Leucine-rich repeat-containing G-protein coupled receptor 5), MEIS3 (Meis homeobox 3), and SMOC2 (SPARC related modular calcium binding 2). Conversely, mRNA levels of LPX (leupaxin) and IGF2BP3 (insulin-like growth factor 2-binding protein 3) were increased by AA (Figure 5B lower plot).

Enrichment analysis by Gene Ontology (GO) GeneSet assessment identified relevant pathways (with *p* < 0.01) involving the genes differentially expressed in AA and CM conditions by outgrowing (Table S3) and expanded OB populations (Table S4). Among the most highly ranked pathways for expanded OB were several pathways involved in apoptosis and cell death, as well as mitotic cell cycle arrest and catabolic processes (Figure 5C, Table S4). One of the pathways related to apoptosis was selected and is shown as an example of gene expression distribution within this pathway (Figure 5D). At single gene level, only the expression of TNFRSF21 (tumor necrosis factor receptor superfamily member 21, see also Table S2) and IL1A (interleukin 1A, not listed in Table S2 since the log2 fold change was <1.5) were identified to be significant (*adjusted p* < 0.05). All other identified pathways are depicted and labelled in Supplemental Figure S1.

## 3. Discussion

While increasing evidence highlights the importance of cellular senescence in the development of OA [20], there is still a lack of studies investigating senescence of human osteoarthritic osteoblasts. In this study, we showed that human primary osteoblasts, isolated from osteoarthritic subchondral bone, become senescent in vitro and that this can be attenuated by the application of an antioxidant. We demonstrated a beneficial effect of AA application to primary osteoarthritic OB, yielding an increased number of outgrowing cells with superior proliferative capacity. Together, these effects result in cell numbers several times higher than at standard culture conditions. The cellular characteristics of OB, regarding for instance the expression profiles of OC and Col1, were not affected under the conditions here applied; on the contrary, we demonstrated an antisenescence effect of AA on replicative senescence of osteoarthritic OB, most likely via a reduction of oxidative stress. The bone forming capacity, however, remained unaltered in the presence of AA in our conditions. 

Since, despite maintaining the osteoblastic phenotype of the cells, AA had a significant effect on cell proliferation and cellular senescence, transcriptomic analyses were conducted to unravel the entailed molecular alterations. Selected genes that were expressed at significantly higher levels at control conditions included PTX3, IGFBP1, and SESN2. PTX3 has been described in the literature to bind to apoptotic cells thus regulating their clearance [40], while IGFBP1, which regulates the IGF signalling pathway, has been described to inhibit proliferation and cell survival of bone marrow-derived mesenchymal stem cells [41]. SESN2 is a known stress responsive molecule, buffering toxic ROS levels [42]. Transcripts of the stress-regulating protein HAS1 [43] were upregulated by AA, and, interestingly, HAS1 deficiency has been correlated with chronic inflammation and cartilage degeneration in mouse models [44]. Following monolayer expansion of OB, transcript levels of LGR5 and MEIS3 were high at control conditions, whereas LPX and IGF2BP3 levels were reduced following AA supplementation. LGR5 is a receptor mediating Wnt signalling, an important pathway involved in development and tissue homeostasis but also known to mediate cellular senescence [45,46]. Changes in the Wnt/β-catenin signalling pathway deriving from aging may result in reduced cellularity and scarcity of progenitor cells, leading to a reduced regenerative capacity of the articular cartilage, which contributes to OA development [47]. Besides a function in regulating cell cycling, typical of IGF-binding proteins [48], IGF2BP3 was found to be involved in RNA synthesis and metabolism, through the destabilisation of MEIS3 mRNA [49]. 

Enriched downstream gene analysis for outgrowing OB identified some of the most affected pathways, i.e., the regulation of mesoderm development, cytokine secretion, and polysaccharide binding. In contrast, following expansion, the majority of the identified pathways affected by AA supplementation were involved in apoptotic and catabolic processes, confirming the macroscopically observed attenuation of the onset of senescence and the arrest of mitotic cell cycling.

Of further note is that none of the well-known markers for OB [50] were among those differentially expressed genes, confirming our qPCR observations and suggesting that the specific expression profile of isolated OB is not directly modulated under the conditions here applied. While the outgrowing OB (at P0) showed similar expression profiles irrespective of the culture conditions, with only few differentially expressed genes, OB expanded at P2 could not be well discriminated from P4-expanded OB. A possible explanation of the homologous appearance of P2- and P4-expanded OB could be the temporarily different onset of senescence that varied reasonably between donors. While for some donors high numbers of senescent cells were already detected at P2, (e.g., donor 3), other donors (e.g., donor 1) displayed low senescent levels at P2 and only reached a comparably high level of β-galactosidase-positive cells at P4. This high interdonor and temporal variabilities for the onset of senescence could perhaps explain the high similarity between P2- and P4-expanded OB, while such phenotypic changes could also have been induced by the rather artificial 2D monolayer culture.

AA is a well-known, potent antioxidant, reducing oxidative stress by directly scavenging free radicals as well as by restoring the antioxidative properties of other factors, such as vitamin E [51]. As a consequence, the accumulation of ROS decreases, and cells and tissues are protected against excessive ROS production that may induce cellular death [52,53]. Our observed decrease of ROS expression is most likely mediated by AA, providing an antioxidant defence to maintain cell viability and cell proliferative capacity by reduced oxidative stress.

Little is known about the regulation of antioxidant enzymes in OA and their transportation from the circulation to the tissues. At present, the supply of nutrients, oxygen, and antioxidants to the cartilage-residing chondrocytes is thought to occur by diffusion from subchondral bone and the synovial microcirculation [54,55]. Chondrocytes were shown to generate abnormal levels of ROS in response to oxygen variations [56]—induced through ischemia–reperfusion injury, mechanical stress, immunomodulatory and inflammatory mediators—and high levels of nitric oxide (NO) in response to proinflammatory cytokines [57]. NO has been implicated in OA and described to inhibit extracellular matrix production by interfering with important autocrine and paracrine factors [58]. Still, precise mechanisms and the role of these factors in the communication between cells that reside in the different synovial joint tissues remain poorly understood and require more studies to unravel their function in health and disease. The poorly understood cross-talk of bone and cartilage-residing cells can be explored in vitro in co-culture models, which already revealed the capacity of OA-OB to modulate the chondrocytic phenotype [14,15]. Whether these intercellular interactions are altered when the number of senescent cells is modified, such as, as shown here, in the presence of AA, would need to be tested in defined co-culture settings, and are indeed currently under investigation in our group.

The main limitation of this study is the lack of healthy human OB because of the lack of availability of such biopsy samples. Such comparative analysis of OB from healthy versus OA bone could give interesting insights into the cellular changes occurring in disease and indicate whether the here observed cellular senescence is a common feature of OB in vitro, or a rather specific characteristic of OB from OA joints. It would also be of interest to investigate whether protection from oxidative stress could rejuvenate the OB phenotype by shifting the expression profile of diseased OB towards the one of healthy OB. A possible mechanism for such cell rejuvenation could be the attenuation of the telomere shortening rate, as demonstrated for fibroblasts upon AA application [59]. Alternatively, telomerase activation could be targeted with phytochemicals that demonstrated to rejuvenate ageing cells by counteracting senescence symptoms [60,61]. For mesenchymal stromal/stem cells (MSCs), telomere shortening was counteracted by hTERT transduction, resulting in prolonged replicative capacity in vitro and maintenance of their multilineage differentiation potential [62,63,64]. However, the clinical relevance of generating large numbers of MSCs remains unclear and needs further investigation. Future studies should aim to identify factors preventing MSC-derived chondrocytes and osteoblasts from undergoing premature senescence and to understand how to maintain them in their healthy state [65].

Under our culture conditions, we could not reproduce the previously described differential mineralisation capacity of Sc_OB and N_OB [66]. A possible explanation could be that our biopsies, which were derived from patients undergoing total knee joint replacement, were already at rather late stages of joint degeneration, and thus physiological and cellular changes due to OA had already affected the mineralisation capacity of cells in the entire joint. While the marrow population was removed from the trabecular bone via digestion, the purity of the cell population claimed to be OB remains unclear. Most likely, the cell population here analysed consisted of a heterogenous pool containing OB and mesenchymal stromal progenitor cells, e.g., bone marrow-derived osteogenic progenitor cells identified to express ALP [67], or myeloid cells expressing ALP and OC [68]. For further investigations, a more defined and homogenous cell population, possibly deriving from single cells, could be addressed.

Since the senolytic clearance of senescent cells in cartilage was recently shown to delay the onset of OA [32], the potential application of antisenescent interventions seems to be a promising therapeutic approach for prevention or even treatment of OA. Here, we demonstrated that also the bone-residing cells of an OA joint are susceptible to senescence, most likely decreasing the capacity to maintain tissue homeostasis in the joint. The specific prevention of senescence in joint cells could attenuate ageing processes and result in a better maintenance of tissue functionality. Targeting cellular senescence could thus present a novel therapeutic approach to treat, delay, or even prevent OA or other age-related diseases.

Whether the reduction of oxidative stress is the proper approach in the quest to find a suitable therapeutic for OA, or whether the senolytic clearance of specific cell types, or even the inhibition of the secretion of senescence-associated factors by senescent cells are required, will need to be investigated in more detail with a larger cohort of samples and in OA animal models.

Our here presented data, showing an improvement of OB culture conditions by supplementation with AA, can also help to achieve sufficient cell numbers in vitro in preclinical studies for the systematic investigation of OB, for example regarding their interactions with chondrocytes in OA development.

## 4. Materials and Methods

### 4.1. Isolation and Expansion of Human Osteoarthritic Osteoblasts

All subjects gave their informed consent for inclusion before they participated in the study. The study was conducted in accordance with the Declaration of Helsinki, and the protocol was approved by the Ethics Committee of Basel (No.147/12). Human subchondral bone biopsies were obtained from *n* = 15 patients with osteoarthritis (OA) (12 female, 3 male; age range from 40–89 years with a mean of 71.5 years) undergoing total knee replacement. Sclerotic and non-sclerotic regions within the same joint were identified by visual inspection, with the sclerotic subchondral bone plate defined by its thickness of ≥2 mm and being covered with debrided or denuded cartilage, while non-sclerotic bone regions were ≤1 mm in thickness and covered by healthy-looking hyaline cartilage. Areas of approximately 1 cm^2^ were cut out with a bone saw, and cartilage was separated from the subchondral bone unit (cortical plate and trabecular bone) with a scalpel. Bone plates were further cut in chips of 1 mm thickness with a metal scalpel. To remove marrow tissue from the trabecular bone, bone chips were washed thoroughly before digestion in 0.6 mg/mL clostidrial collagenase type IA (Sigma Aldrich, St. Louis, MO, USA) for 120 min (similarly as described by [69]). The weight of each bone chip was determined before placing it into one well of a six-well plate with standard culture medium (CM = Dulbecco’s modified medium, DMEM containing 10% fetal bovine serum, 10 mM HEPES, 1 mM sodium pyruvate, 100 U/mL penicillin, 100 µg/mL streptomycin, 0.29 mg/mL l-glutamine) as such or with additional 0.1 mM ascorbic acid 2-phosphate (Sigma; +AA). Each experimental condition was set up in triplicates (technical replicates). The medium was changed twice a week. At subconfluency after 7 days, outgrowing OBs (=passage P0) were detached using 0.05% trypsin/0.53 mM EDTA (ThermoFisher Scientific, Waltham, MA, USA) and counted. For outgrowth efficiency, cell numbers were normalised to the corresponding bone weight. For expansion up to P4–P5, OB were serially trypsinised at 70–80% confluency, counted and reseeded (in duplicates) at a cell density of 10^5^ cells/cm^2^ into six-well plates for expansion in CM with or without AA. The proliferation rate was determined by the number of population doublings per day, and the life span defined by the number of accumulated population doubling until cellular senescence of at least 90% of the cells.

### 4.2. Surface Marker Expression

The expression of alkaline phosphatase (ALP) and osteocalcin (OC) in primary osteoblasts were determined by flow cytometry as described previously [69]. Antibodies for flow cytometry were PerCP–Cy5.5-conjugate anti-alkaline phosphatase (Clone B4-78, BD Pharmingen, Allschwil, Switzerland). Osteocalcin was stained by unconjugated rabbit anti-osteocalcin antibodies (AB1857, Merck, Burlington, MA, USA), followed by Atto 550-conjugated goat anti-rabbit-IgG (43328, Sigma). Approximately 1 × 10^5^ OB were transferred per polystyrene FACS tube and centrifuged (220 g, 3 min). After 30 min incubation on ice and following washing, cells were incubated with 5 µL of Human TruStain FcX (Fc Receptor Blocking Solution, BioLegend, San Diego, CA, USA) in 100 µL for 10 min on ice to minimise nonspecific antibody binding. OB were first stained with the anti-alkaline phosphatase antibody for 30 min on ice, prior to washing and incubation in fixation–permeabilisation buffer for 15 min on ice as per the manufacturer’s instructions (R&D Systems, Minneapolis, MN, USA). Cells were centrifuged and the supernatant removed until approximately 100 µL of fixation–permeabilization buffer remained. Osteocalcin staining was then performed for 30 min on ice, followed by washing, resuspension, and staining in 1 mL FACS buffer containing 4 µL of Atto 550-conjugated secondary antibody for 30 min on ice. After staining, all cell-containing tubes were washed once more with 1 mL of FACS buffer, centrifuged, and resuspended in 300 µL of FACS buffer for analysis. All FACS analyses were performed in a BD LSRFortessa (Becton Dickinson, Franklin Lakes, NJ, USA) cell analyser.

### 4.3. Immunohistochemistry

Fixed cells were stained in the six-well plates with rabbit anti-human Ki67 antibodies (1:200, Abcam 15580, Cambridge, UK), overnight at 4 °C. Following incubation with the secondary anti-rabbit Alexa Fluor 488 antibody (1:400, Abcam 150088) for 1h in the dark, the cells were counterstained with 300 nM 4′,6-diamidino-2-phenylindole (DAPI, ThermoFisher Scientific) for 30 min.

### 4.4. Quantitative Real-Time Reverse Transcriptase PCR

Total RNA was isolated with Quick-RNA Mini Prep from ZYMO Research, and the cDNA synthesised from 300 ng RNA with random primers (Promega, Madison, WI, USA) and SuperScript reverse Transcriptase III (Thermo Fisher, Waltham, MA, USA), as recommended by the manufacturer [70]. Transcript quantification was performed in duplicates using a GeneAmp PCR system and TaqMan^TM^ gene detection assays from Thermofisher Scientific. The results were normalised against the expression of the house-keeping gene *GAPDH*.

### 4.5. Identification and Quantification of Senescent Cells

Using the Senescence Detection Kit (#K320-250, BioVision, Milpitas, CA, USA), senescence associated β-galactosidase expression (SA-β-Gal) was detected for Sc_OB and N_OB for each passage from P0 to P4 at subconfluency, according to the manufacturer’s instructions. Briefly, cells were washed and fixed for 15 min in the provided Fixative Solution at room temperature. Following the removal of the Fixative Solution and after washing, the cells were incubated overnight at 37 °C with a freshly prepared staining solution, consisting of Staining Solution (940 µL/mL), Staining Supplement (10 µL/mL), and 1 mg/mL X-Gal. Senescent cells were positively stained in blue, and their number determined on pictures of four randomly selected regions at 10× magnification and counted by two blinded operators. The percentage of senescent cells was related to the total number of cells, identified following a counterstaining with Nuclear Fast Red for 15 min or following a staining of nuclei with 300 nM DAPI for 30 min.

### 4.6. Quantification of Reactive Oxygen Species (ROS)

For the quantification of intracellular ROS production, 1 × 10^5^ cells in pre-warmed PBS were incubated with 10 µM 2′,7′-dichlorodihydrofluorescein (H_2_DCF; Sigma-Aldrich D6883-50MG, St. Louis, MO, USA) for 30 min at 37 °C. The oxidation of H_2_DCF to dichlorofluorescein (DCF) was measured by flow cytometry as the fluorescence intensity at 488 nm excitation–535 nm emission. 

### 4.7. Osteogenic Differentiation

Osteogenic differentiation was induced in six-well plates for P2-expanded OB (seeding density: 5 × 10^3^ cells/cm^2^) using osteogenic medium (OM = standard culture medium as described above but prepared in αMEM (instead of DMEM), and additionally supplemented with 10 nM dexamethasone, 10 mM β-glycerophosphate, and 0.1 mM AA—all from Sigma) for up to 21 days, according to standard protocols [71]. The respective control medium lacked dexamethasone and β-glycerophosphate. Mineralisation was visualised by staining formalin-fixed monolayers in 2% alizarin red for 10 min as previously described [71].

### 4.8. In Vivo Implantation of Osteogenic Constructs

After isolation and expansion up to P2, 3–4.5 × 10^3^ OB/mm^3^ were seeded on a ceramic Engipore scaffold (Finceramica, Italy, 6 mm diameter, 4 mm high) and pre-cultured in osteogenic medium (OM, see osteogenic differentiation) for 14 days, with the medium changed twice a week. OB-seeded constructs were implanted subcutaneously in nude mice (Nu/Nu, Charles-River, Sulzfeld, Germany), as previously described [72]. Two constructs were implanted for each condition and explanted 4 weeks later. The animals were treated in agreement with the Swiss legislation and according to a protocol approved by the Veterinary Office of Canton Basel-Stadt (permission # 1797).

### 4.9. Histological Processing

The explanted constructs were washed, fixed for 8 h with 1% paraformaldehyde, and decalcified in PBS containing 7% *w*/*v* EDTA and 10% *w*/*v* sucrose at 37 °C on an orbital shaker, according to established protocols [73]. The renewal of the decalcification solution was performed daily for approximately 2 weeks, until the samples were fully decalcified, as estimated by the degree of sample stiffness. The samples were embedded in OCT compound (Sakura Finetek, Torrance, CA, USA) and frozen in isopentane for cryosectioning of 10 µm thick sections with a cryotome. 

### 4.10. Analysis of Bone Formation In Vivo

For the quantitative detection of bone tissue, slides were stained with haematoxylin and eosin (H&E) and microscopically observed under both transmitted and fluorescent light (excitation 546 nm; emission 590 nm), as previously described [74]. In addition, the presence of bone matrix was examined with Masson trichrome staining (Réactifs RAL, Martillac, France), performed according to the manufacturer’s instructions. The areas covered with bone-like tissue and the total areas were quantified by computerised image analysis using ImageJ (National Institutes of Health, Bethesda, MD, USA) [75].

### 4.11. Statistical Analyses

All data were presented as mean values ± SD (standard deviation). Using the statistical analysis software GraphPad Prism (GraphPad Software, Inc., San Diego, CA, USA), Mann Whitney-U testing for non-parametric un-paired sample sets was performed if not otherwise mentioned. Non-parametric analysis for paired samples, Wilcoxon, was performed for the analysis of the total population doublings. For each donor and experimental group, technical duplicates or triplicates were analysed; *p* values *<* 0.05 were considered significant.

### 4.12. Transcriptomic Analyses

Following outgrowth (P0) and expansion for two or four passages (P2 and P4, respectively), 0.2–0.5 × 10^6^ OB (Sc_OB or N_OB) of *n* = 4 donors were processed for transcriptomic analysis.

*Library preparation:* total RNA was isolated using Quick-RNA^TM^ MiniPrep Kit (Zymo Research, #R1055, Irvine, CA, USA) according to the manufacturer’s instructions. RNA quality was checked with the Bioanalyzer instrument (Agilent Technologies, Santa Clara, CA, USA) using the RNA 6000 Pico Chip (Agilent, Cat# 5067-1513) and quantified by Fluorometry using the QuantiFluor RNA System (Cat# E3310, Promega, Madison, WI, USA). Library preparation was performed starting from 100 ng of total RNA (exception: 82 ng for OB4+AA_P0) using the TruSeq Stranded mRNA Library Prep Kit High Throughput (Cat# RS-122-2103, Illumina, San Diego, CA, USA). Libraries were quality checked on the Fragment Analyzer (Advanced Analytical, Ames, IA, USA) using the Standard Sensitivity NGS Fragment Analysis Kit (Cat# DNF-473, Advanced Analytical), revealing an excellent quality of libraries (average concentration was 72 ± 12 nmol/L (or 15 ± 5 ng/µL for OB4+AA_P0); the average library size was 333 ± 9 base pairs. The samples were pooled to equal molarity. Each pool was quantified by PicoGreen Fluorometric measurement in order to be adjusted to 1.8 pM, and used for clustering in the NextSeq 500 instrument (Illumina).

*Clustering and Sequencing:* Samples were sequenced with Single-reads of 76 bases using the NextSeq 500 High Output Kit 75-cycles (Illumina, Cat# FC-404-1005), and primary data analysis was performed with the Illumina RTA version 2.4.11 and Basecalling Version bcl2fastq-2.19.1.403.

*Bioinformatic analysis:* the obtained reads were mapped to the human genome assembly, version hg38, with RNA-STAR [76], with default parameters except for reporting for multimappers only one hit in the final alignment files (outSAMmultNmax = 1), and filtering reads without evidence in spliced junction table (outFilterType = “BySJout”). Using KnownGenes mRNA coordinates from UCSC (https://genome.ucsc.edu, downloaded in April 2017, University of California, Santa Cruz, CA, USA) and the qCount function from QuasR package [77] (version 1.16.0, R version 3.4.0, Bioconductor, Fred Hutchinson Cancer Research Center, Seattle, WA, USA), we quantified gene expression as the number of reads that started within any annotated exon of a gene. The differentially expressed genes were identified using the edgeR package [78] (version 3.18.1, Bioconductor). Genes with FDR (false discovery rate–adjusted *p*-value) smaller than 0.05, and minimum log2 fold-change of ± 1.5 were used for downstream analysis.

## Supplementary Materials

Supplementary materials can be found at www.mdpi.com/1422-0067/18/12/2517/s1.
