# Supplementary material for: Ascorbic Acid Attenuates Senescence of Human Osteoarthritic Osteoblasts
Source: Int J Mol Sci. 2017 Nov 24;18(12):2517. doi: 10.3390/ijms18122517 (PMC5751120; doi:10.3390/ijms18122517)
Supplement: Supplementary file 1 [file ijms-18-02517-s001.pdf]

International Journal of
Molecular Sciences

MDPI

Article

Ascorbic Acid Attenuates Senescence of Human Osteoarthritic Osteoblasts

Maximilian G. Burger [1,2,†], Amir Steinitz [1,3,†], Jeroen Geurts [4], Benjamin E. Pippenger [4], Dirk J. Schaefer [2], Ivan Martin [1], Andrea Barbero [1,*] and Karoliina Pelttari [1]

1 Department of Biomedicine, University of Basel, University Hospital of Basel, 4031 Basel, Switzerland; maximilian.burger@usb.ch (M.G.B.); amir.steinitz@usb.ch (A.S.); ivan.martin@usb.ch (I.M.); karoliina.pelttari@usb.ch (K.P.)
2 Department of Plastic, Reconstructive, Aesthetic and Hand Surgery, University Hospital of Basel, University of Basel, 4031 Basel, Switzerland; dirk.schaefer@usb.ch
3 Departments for Orthopedic Surgery and Traumatology, University Hospital of Basel, 4031 Basel, Switzerland
4 Departments Spine Surgery and Biomedical Engineering, University Hospital of Basel, University of Basel, 4031 Basel, Switzerland; jeroen.geurts@usb.ch (J.G.); benjamin.pippenger@straumann.com (B.E.P.)
* Correspondence: andrea.barbero@usb.ch; Tel.: +41-61-265-2384
† These authors contributed equally to this work.

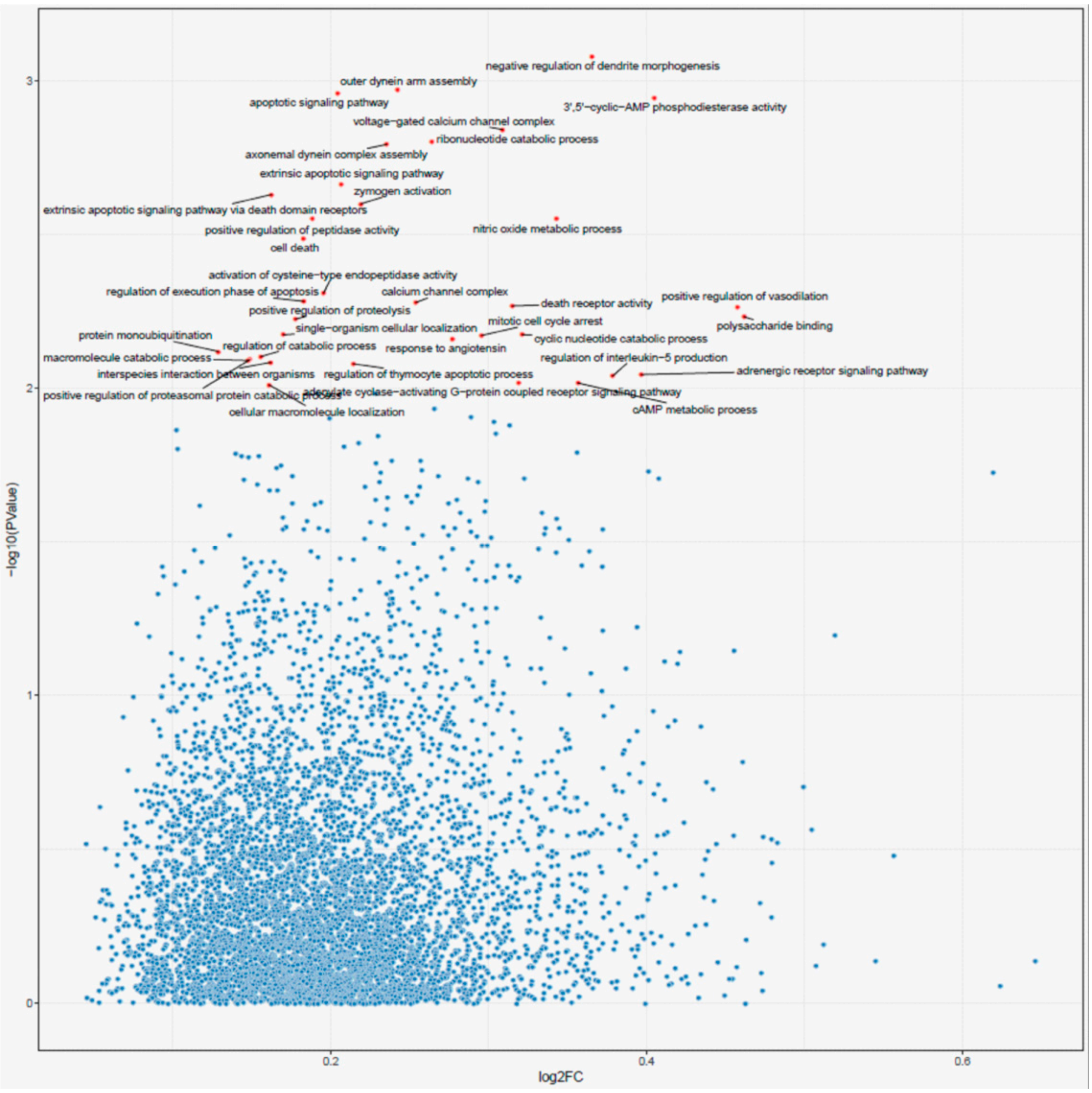

Figure S1. Main pathways affected by AA supplementation in expanded OB, as identified by GO GeneSet downstream analysis, are shown in this scatter plot with the most affected ones ($p < 0.01$) depicted in red and labelled.

Table S1. Genes differentially expressed by osteoarthritic osteoblasts at P0 (outgrowth) cultured with AA, as compared to standard culture medium alone, which showed at least 1.5-fold higher (positive values) or lower expression (negative values); $p < 0.05$; genes in *italic* (above the dashed line) have an *adjusted p-value* < 0.05 (FDR, false discovery rate).

Entrez ID	Symbol	Gene Name	Log2fc	Log2a	*p* Value	Adj.*p*.Val
4622	*MYH4*	*myosin heavy chain 4*	3.54	0.71	5.83×10^{-17}	1.19×10^{-12}
5806	*PTX3*	*pentraxin 3*	−3.77	10.42	1.30×10^{-07}	4.77×10^{-04}
153201	*SLC36A2*	*solute carrier family 36 member 2*	1.90	−1.30	1.40×10^{-07}	4.77×10^{-04}
55801	*IL26*	*interleukin 26*	−2.29	1.40	9.52×10^{-07}	2.77×10^{-03}
84870	*RSPO3*	*R-spondin 3*	−2.42	2.01	6.24×10^{-06}	1.14×10^{-02}
205	*AK4*	*adenylate kinase 4*	−2.22	5.75	7.27×10^{-06}	1.14×10^{-02}
12	*SERPINA3*	*serpin family A member 3*	−2.17	1.37	8.95×10^{-06}	1.26×10^{-02}
56241	*SUSD2*	*sushi domain containing 2*	−2.54	3.94	9.25×10^{-06}	1.26×10^{-02}
8482	*SEMA7A*	*semaphorin 7A (John Milton Hagen blood group)*	1.63	7.36	1.01×10^{-05}	1.29×10^{-02}
131578	*LRRC15*	*leucine rich repeat containing 15*	2.08	6.07	1.52×10^{-05}	1.72×10^{-02}
7857	*SCG2*	*secretogranin II*	−2.20	1.56	1.61×10^{-05}	1.73×10^{-02}
1356	*CP*	*ceruloplasmin*	−2.34	2.27	1.85×10^{-05}	1.89×10^{-02}
81539	*SLC38A1*	*solute carrier family 38 member 1*	−3.05	5.72	2.24×10^{-05}	2.14×10^{-02}
100505633	*LINC01133*	*long intergenic non-protein coding RNA 1133*	−1.69	4.65	2.88×10^{-05}	2.51×10^{-02}
55714	*TENM3*	*teneurin transmembrane protein 3*	1.64	4.47	2.95×10^{-05}	2.51×10^{-02}
23566	*LPAR3*	*lysophosphatidic acid receptor 3*	1.52	−0.38	3.76×10^{-05}	3.01×10^{-02}
7164	*TPD52L1*	*tumor protein D52 like 1*	−2.19	5.57	3.83×10^{-05}	3.01×10^{-02}
84281	*C2orf88*	*chromosome 2 open reading frame 88*	1.87	2.11	4.08×10^{-05}	3.08×10^{-02}
7226	*TRPM2*	*transient receptor potential cation channel subfamily M member 2*	1.65	−0.53	4.34×10^{-05}	3.08×10^{-02}
3484	*IGFBP1*	*insulin like growth factor binding protein 1*	−1.64	3.09	4.37×10^{-05}	3.08×10^{-02}
2687	*GGT5*	*gamma-glutamyltransferase 5*	−2.01	3.91	5.67×10^{-05}	3.62×10^{-02}
3036	*HAS1*	*hyaluronan synthase 1*	2.28	2.88	6.61×10^{-05}	4.08×10^{-02}
5308	*PITX2*	*paired like homeodomain 2*	1.71	−0.57	7.54×10^{-05}	4.28×10^{-02}
83667	*SESN2*	*sestrin 2*	−1.77	5.36	7.55×10^{-05}	4.28×10^{-02}
56901	NDUFA4L2	NDUFA4, mitochondrial complex associated like 2	−3.41	5.04	9.85×10^{-05}	5.31×10^{-02}
26471	NUPR1	nuclear protein 1, transcriptional regulator	−1.67	6.66	1.22×10^{-04}	5.67×10^{-02}
6480	ST6GAL1	ST6 beta-galactoside alpha−2,6-sialyltransferase 1	−2.07	2.18	1.22×10^{-04}	5.67×10^{-02}
64094	SMOC2	SPARC related modular calcium binding 2	−2.06	2.74	1.22×10^{-04}	5.67×10^{-02}
55638	SYBU	syntabulin	−2.20	0.99	1.30×10^{-04}	5.91×10^{-02}
80329	ULBP1	UL16 binding protein 1	−1.75	3.65	1.43×10^{-04}	6.34×10^{-02}
4620	MYH2	myosin heavy chain 2	2.67	−0.20	1.47×10^{-04}	6.37×10^{-02}
286133	SCARA5	scavenger receptor class A member 5	−2.07	2.28	1.74×10^{-04}	6.97×10^{-02}

Table S1. *Cont.*

Entrez ID	Symbol	Gene Name	Log2fc	Log2a	*p* Value	Adj.*p*.Val
93145	OLFM2	olfactomedin 2	−2.08	4.28	1.99×10^{-04}	7.55×10^{-02}
4883	NPR3	natriuretic peptide receptor 3	−1.86	7.66	2.69×10^{-04}	8.86×10^{-02}
1082	CGB3	chorionic gonadotropin beta subunit 3	1.77	−1.44	2.80×10^{-04}	8.91×10^{-02}
9249	DHRS3	dehydrogenase/reductase 3	−2.03	4.15	2.93×10^{-04}	8.91×10^{-02}
55966	AJAP1	adherens junctions associated protein 1	1.63	0.60	2.97×10^{-04}	8.91×10^{-02}
378706	RN7SL2	RNA, 7SL, cytoplasmic 2	−1.62	6.28	3.45×10^{-04}	9.76×10^{-02}
79094	CHAC1	ChaC glutathione specific gamma-glutamylcyclotransferase 1	−2.18	4.57	3.71×10^{-04}	1.04×10^{-01}
5104	SERPINA5	serpin family A member 5	−1.81	0.82	4.03×10^{-04}	1.07×10^{-01}
401491	VLDLR-AS1	VLDLR antisense RNA 1	−1.65	−0.05	4.04×10^{-04}	1.07×10^{-01}
79924	ADM2	adrenomedullin 2	−2.43	4.18	4.41×10^{-04}	1.11×10^{-01}
3485	IGFBP2	insulin like growth factor binding protein 2	−2.90	7.65	4.58×10^{-04}	1.13×10^{-01}
116039	OSR2	odd-skipped related transciption factor 2	−1.84	3.13	4.95×10^{-04}	1.18×10^{-01}
5507	PPP1R3C	protein phosphatase 1 regulatory subunit 3C	−1.53	6.25	6.15×10^{-04}	1.39×10^{-01}
50614	GALNT9	polypeptide N-acetylgalactosaminyltransferase 9	1.58	−0.19	8.00×10^{-04}	1.72×10^{-01}
57528	KCTD16	potassium channel tetramerization domain containing 16	−1.54	3.72	1.09×10^{-03}	2.14×10^{-01}
11341	SCRG1	stimulator of chondrogenesis 1	−2.28	6.09	1.16×10^{-03}	2.18×10^{-01}
1002	CDH4	cadherin 4	2.03	0.97	1.33×10^{-03}	2.30×10^{-01}
9966	TNFSF15	TNF superfamily member 15	−2.49	3.48	1.34×10^{-03}	2.30×10^{-01}
1825	DSC3	desmocollin 3	−1.56	1.86	1.49×10^{-03}	2.45×10^{-01}
79689	STEAP4	STEAP4 metalloreductase	−3.10	4.46	1.56×10^{-03}	2.51×10^{-01}
4619	MYH1	myosin heavy chain 1	2.74	−0.23	2.34×10^{-03}	3.12×10^{-01}
10529	NEBL	nebulette	−2.15	1.33	2.48×10^{-03}	3.17×10^{-01}
60676	PAPPA2	pappalysin 2	−2.63	3.44	2.50×10^{-03}	3.17×10^{-01}
200150	PLD5	phospholipase D family member 5	1.74	−0.72	2.53×10^{-03}	3.18×10^{-01}
11185	INMT	indolethylamine N-methyltransferase	−1.55	1.11	2.67×10^{-03}	3.29×10^{-01}
9507	ADAMTS4	ADAM metallopeptidase with thrombospondin type 1 motif 4	1.96	1.77	3.05×10^{-03}	3.54×10^{-01}
8516	ITGA8	integrin subunit alpha 8	−1.82	3.75	3.21×10^{-03}	3.64×10^{-01}
55244	SLC47A1	solute carrier family 47 member 1	−1.79	1.81	3.28×10^{-03}	3.70×10^{-01}
10653	SPINT2	serine peptidase inhibitor, Kunitz type 2	−1.73	3.85	3.50×10^{-03}	3.84×10^{-01}
57761	TRIB3	tribbles pseudokinase 3	−1.92	5.98	3.61×10^{-03}	3.89×10^{-01}
23657	SLC7A11	solute carrier family 7 member 11	−1.70	6.28	3.78×10^{-03}	3.97×10^{-01}
1306	COL15A1	collagen type XV alpha 1 chain	−2.63	7.31	3.85×10^{-03}	4.03×10^{-01}
100507632	LINC00968	long intergenic non-protein coding RNA 968	−1.70	5.56	4.62×10^{-03}	4.29×10^{-01}
445	ASS1	argininosuccinate synthase 1	−1.56	6.03	4.91×10^{-03}	4.29×10^{-01}
170689	ADAMTS15	ADAM metallopeptidase with thrombospondin type 1 motif 15	−1.58	3.78	5.10×10^{-03}	4.34×10^{-01}

Table S1. *Cont.*

Entrez ID	Symbol	Gene Name	Log2fc	Log2a	*p* Value	Adj.*p*.Val
80763	SPX	spexin hormone	−1.50	2.72	5.61×10^{-03}	4.58×10^{-01}
6299	SALL1	spalt like transcription factor 1	1.85	0.33	5.99×10^{-03}	4.70×10^{-01}
94115	CGB8	chorionic gonadotropin beta subunit 8	1.97	−0.01	6.04×10^{-03}	4.70×10^{-01}
3911	LAMA5	laminin subunit alpha 5	−1.73	4.32	6.36×10^{-03}	4.70×10^{-01}
7018	TF	transferrin	−1.61	−0.13	8.04×10^{-03}	4.97×10^{-01}
10335	MRVI1	murine retrovirus integration site 1 homolog	−1.81	5.11	8.75×10^{-03}	5.09×10^{-01}
2202	EFEMP1	EGF containing fibulin like extracellular matrix protein 1	−1.72	10.45	8.81×10^{-03}	5.09×10^{-01}
7477	WNT7B	Wnt family member 7B	2.10	2.76	9.07×10^{-03}	5.14×10^{-01}
50509	COL5A3	collagen type V alpha 3 chain	−1.54	7.39	9.23×10^{-03}	5.16×10^{-01}
93659	CGB5	chorionic gonadotropin beta subunit 5	1.75	−1.08	9.78×10^{-03}	5.27×10^{-01}
151354	FAM84A	family with sequence similarity 84 member A	1.56	0.41	1.17×10^{-02}	5.48×10^{-01}
2331	FMOD	fibromodulin	−1.72	5.69	1.17×10^{-02}	5.48×10^{-01}
1300	COL10A1	collagen type X alpha 1 chain	1.60	4.19	1.37×10^{-02}	5.77×10^{-01}
3696	ITGB8	integrin subunit beta 8	−1.62	4.69	1.43×10^{-02}	5.82×10^{-01}
8728	ADAM19	ADAM metallopeptidase domain 19	1.77	5.88	1.43×10^{-02}	5.82×10^{-01}
1675	CFD	complement factor D	−2.26	1.76	1.44×10^{-02}	5.82×10^{-01}
196051	PLPP4	phospholipid phosphatase 4	1.52	0.57	1.71×10^{-02}	6.19×10^{-01}
1612	DAPK1	death associated protein kinase 1	−1.51	5.62	2.06×10^{-02}	6.50×10^{-01}
5055	SERPINB2	serpin family B member 2	1.71	5.20	2.11×10^{-02}	6.50×10^{-01}
83643	CCDC3	coiled-coil domain containing 3	1.50	2.20	2.63×10^{-02}	6.94×10^{-01}
4232	MEST	mesoderm specific transcript	−1.56	7.12	2.64×10^{-02}	6.95×10^{-01}
150	ADRA2A	adrenoceptor alpha 2A	−1.93	4.20	2.75×10^{-02}	6.98×10^{-01}
4804	NGFR	nerve growth factor receptor	1.55	0.98	2.90×10^{-02}	7.03×10^{-01}
1101	CHAD	chondroadherin	2.55	0.00	3.18×10^{-02}	7.16×10^{-01}
1758	DMP1	dentin matrix acidic phosphoprotein 1	1.91	−0.83	3.32×10^{-02}	7.29×10^{-01}
4915	NTRK2	neurotrophic receptor tyrosine kinase 2	−1.77	4.25	3.40×10^{-02}	7.34×10^{-01}
79987	SVEP1	sushi, von Willebrand factor type A, EGF and pentraxin domain containing 1	−1.76	6.09	3.88×10^{-02}	7.63×10^{-01}
84929	FIBCD1	fibrinogen C domain containing 1	1.51	1.84	4.10×10^{-02}	7.78×10^{-01}
6414	SELENOP	selenoprotein P	−2.07	5.54	4.21×10^{-02}	7.83×10^{-01}
5179	PENK	proenkephalin	−1.93	8.64	4.47×10^{-02}	8.02×10^{-01}
72	ACTG2	actin, gamma 2, smooth muscle, enteric	1.66	4.68	4.99×10^{-02}	8.18×10^{-01}

Table S2. Genes differentially expressed by expanded osteoarthritic osteoblasts (for 2 and 4 passages) cultured with AA, as compared to osteoblasts cultured in standard culture medium alone, which showed at least 1.5-fold higher (positive values) or lower expression (negative values), with $p < 0.05$; genes in *italic* (above the dashed line) have an adjusted *p-value* (FDR, false discovery rate) < 0.05.

Entrez ID	Symbol	Gene Name	Log2fc	Log2a	*p* Value	Adj.*p*.Val
8549	LGR5	*leucine rich repeat containing G protein-coupled receptor 5*	−2.34	2.79	1.32×10^{-18}	2.70×10^{-14}
9404	LPXN	*leupaxin*	1.55	5.03	2.34×10^{-11}	2.39×10^{-07}
56917	MEIS3	*Meis homeobox 3*	−2.35	3.28	8.16×10^{-11}	4.16×10^{-07}
80760	ITIH5	*inter-alpha-trypsin inhibitor heavy chain family member 5*	−3.65	4.72	2.32×10^{-10}	9.47×10^{-07}
56901	NDUFA4L2	*NDUFA4, mitochondrial complex associated like 2*	−3.36	5.04	1.02×10^{-08}	2.61×10^{-05}
57633	LRRN1	*leucine rich repeat neuronal 1*	1.69	2.66	1.34×10^{-08}	3.03×10^{-05}
7225	TRPC6	*transient receptor potential cation channel subfamily C member 6*	−1.70	2.09	1.48×10^{-08}	3.03×10^{-05}
9674	KIAA0040	*KIAA0040*	−1.65	0.61	5.01×10^{-08}	8.52×10^{-05}
773	CACNA1A	*calcium voltage-gated channel subunit alpha1 A*	−1.88	2.54	1.04×10^{-07}	1.62×10^{-04}
1066	CES1	*carboxylesterase 1*	−2.34	1.98	1.27×10^{-07}	1.72×10^{-04}
57221	ARFGEF3	*ARFGEF family member 3*	−2.39	1.70	2.81×10^{-07}	3.37×10^{-04}
1824	DSC2	*desmocollin 2*	−1.51	1.14	3.28×10^{-07}	3.72×10^{-04}
56241	SUSD2	*sushi domain containing 2*	−2.29	3.94	3.87×10^{-07}	4.16×10^{-04}
183	AGT	*angiotensinogen*	−2.02	1.66	6.23×10^{-07}	6.05×10^{-04}
1825	DSC3	*desmocollin 3*	−2.10	1.86	7.20×10^{-07}	6.39×10^{-04}
23467	NPTXR	*neuronal pentraxin receptor*	−1.64	3.57	9.75×10^{-07}	7.96×10^{-04}
205	AK4	*adenylate kinase 4*	−1.60	5.75	1.06×10^{-06}	8.34×10^{-04}
5577	PRKAR2B	*protein kinase cAMP-dependent type II regulatory subunit beta*	−1.50	2.12	1.44×10^{-06}	1.09×10^{-03}
81035	COLEC12	*collectin subfamily member 12*	2.69	5.53	2.40×10^{-06}	1.68×10^{-03}
5228	PGF	*placental growth factor*	−1.56	3.69	2.47×10^{-06}	1.68×10^{-03}
64094	SMOC2	*SPARC related modular calcium binding 2*	−2.76	2.74	8.09×10^{-06}	4.02×10^{-03}
84978	FRMD5	*FERM domain containing 5*	2.09	1.45	9.45×10^{-06}	4.59×10^{-03}
4915	NTRK2	*neurotrophic receptor tyrosine kinase 2*	−2.96	4.25	1.24×10^{-05}	5.49×10^{-03}
8497	PPFIA4	*PTPRF interacting protein alpha 4*	−1.55	3.28	1.30×10^{-05}	5.49×10^{-03}
122402	TDRD9	*tudor domain containing 9*	−2.16	1.97	1.81×10^{-05}	6.71×10^{-03}
60676	PAPPA2	*pappalysin 2*	−2.60	3.44	1.88×10^{-05}	6.85×10^{-03}
55799	CACNA2D3	*calcium voltage-gated channel auxiliary subunit alpha2delta 3*	−2.19	2.42	2.44×10^{-05}	8.43×10^{-03}
80763	SPX	*spexin hormone*	−2.07	2.72	2.85×10^{-05}	9.53×10^{-03}
26033	ATRNL1	*attractin like 1*	−1.88	1.48	3.14×10^{-05}	1.02×10^{-02}
9369	NRXN3	*neurexin 3*	−1.81	4.06	3.65×10^{-05}	1.05×10^{-02}
2890	GRIA1	*glutamate ionotropic receptor AMPA type subunit 1*	−2.12	3.12	3.70×10^{-05}	1.05×10^{-02}
55638	SYBU	*syntabulin*	−1.51	0.99	3.77×10^{-05}	1.05×10^{-02}
57188	ADAMTSL3	*ADAMTS like 3*	−1.74	1.23	4.65×10^{-05}	1.26×10^{-02}

Table S2. *Cont.*

Entrez ID	Symbol	Gene Name	Log2fc	Log2a	*p* Value	Adj.*p*.Val
57569	ARHGAP20	*Rho GTPase activating protein 20*	−2.07	1.22	4.70×10^{-05}	1.26×10^{-02}
27242	TNFRSF21	*TNF receptor superfamily member 21*	1.74	3.76	5.05×10^{-05}	1.34×10^{-02}
2150	F2RL1	*F2R like trypsin receptor 1*	1.60	1.42	6.53×10^{-05}	1.57×10^{-02}
255324	EPGN	*epithelial mitogen*	1.69	1.59	7.16×10^{-05}	1.70×10^{-02}
10643	IGF2BP3	*insulin like growth factor 2 mRNA binding protein 3*	1.52	3.00	7.78×10^{-05}	1.80×10^{-02}
2122	MECOM	*MDS1 and EVI1 complex locus*	1.73	1.83	9.71×10^{-05}	2.11×10^{-02}
79870	BAALC	*brain and acute leukemia, cytoplasmic*	1.68	5.27	1.83×10^{-04}	3.27×10^{-02}
5046	PCSK6	*proprotein convertase subtilisin/kexin type 6*	−1.80	0.12	2.06×10^{-04}	3.46×10^{-02}
286133	SCARA5	*scavenger receptor class A member 5*	−2.03	2.28	2.47×10^{-04}	3.91×10^{-02}
93649	MYOCD	*myocardin*	−1.79	3.75	3.10×10^{-04}	4.62×10^{-02}
340527	NHSL2	*NHS like 2*	2.21	3.40	3.28×10^{-04}	4.78×10^{-02}
2	A2M	alpha-2-macroglobulin	−1.51	3.81	3.72×10^{-04}	5.14×10^{-02}
151449	GDF7	growth differentiation factor 7	−1.86	0.90	4.31×10^{-04}	5.71×10^{-02}
7477	WNT7B	Wnt family member 7B	1.58	2.76	4.41×10^{-04}	5.76×10^{-02}
11341	SCRG1	stimulator of chondrogenesis 1	−1.65	6.09	5.16×10^{-04}	6.34×10^{-02}
55016	Mrz 01	membrane associated ring-CH-type finger 1	−1.51	0.32	5.20×10^{-04}	6.35×10^{-02}
3553	IL1B	interleukin 1 beta	1.58	0.57	5.35×10^{-04}	6.43×10^{-02}
10216	PRG4	proteoglycan 4	−2.13	2.32	5.66×10^{-04}	6.55×10^{-02}
1356	CP	ceruloplasmin	−2.22	2.27	7.62×10^{-04}	7.70×10^{-02}
10234	LRRC17	leucine rich repeat containing 17	1.71	3.47	7.93×10^{-04}	7.97×10^{-02}
3861	KRT14	keratin 14	1.94	7.08	1.02×10^{-03}	9.12×10^{-02}
150	ADRA2A	adrenoceptor alpha 2A	−2.32	4.20	1.09×10^{-03}	9.50×10^{-02}
100133941	CD24	CD24 molecule	−1.76	5.50	1.39×10^{-03}	1.12×10^{-01}
1001	CDH3	cadherin 3	1.91	1.52	1.47×10^{-03}	1.15×10^{-01}
3880	KRT19	keratin 19	1.55	6.21	2.13×10^{-03}	1.44×10^{-01}
125	ADH1B	alcohol dehydrogenase 1B (class I), beta polypeptide	−2.04	1.67	2.30×10^{-03}	1.50×10^{-01}
7148	TNXB	tenascin XB	−1.70	6.90	2.56×10^{-03}	1.61×10^{-01}
63876	PKNOX2	PBX/knotted 1 homeobox 2	−1.59	1.54	2.62×10^{-03}	1.63×10^{-01}
3488	IGFBP5	insulin like growth factor binding protein 5	−1.50	10.53	3.14×10^{-03}	1.81×10^{-01}
23508	TTC9	tetratricopeptide repeat domain 9	−1.57	3.40	3.23×10^{-03}	1.85×10^{-01}
283383	ADGRD1	adhesion G protein-coupled receptor D1	−1.77	1.61	4.44×10^{-03}	2.15×10^{-01}
145270	PRIMA1	proline rich membrane anchor 1	−1.59	0.52	4.95×10^{-03}	2.27×10^{-01}
5064	PALM	paralemmin	−1.58	3.66	5.48×10^{-03}	2.36×10^{-01}
7373	COL14A1	collagen type XIV alpha 1 chain	−1.92	7.99	8.43×10^{-03}	2.96×10^{-01}
5136	PDE1A	phosphodiesterase 1A	−1.59	3.85	1.34×10^{-02}	3.84×10^{-01}

119587	CPXM2	carboxypeptidase X, M14 family member 2	−1.56	3.28	1.55×10^{-02}	4.16×10^{-01}
1306	COL15A1	collagen type XV alpha 1 chain	−1.61	7.31	1.72×10^{-02}	4.39×10^{-01}
79689	STEAP4	STEAP4 metalloreductase	−1.78	4.46	4.71×10^{-02}	6.85×10^{-01}

Table S3. GO GeneSet assessment for the identification of pathways with differentially expressed genes in by expanded osteoarthritic osteoblasts if cultured with AA, as compared to standard culture medium ($p < 0.01$). Pathways beneath the dashed line showed significance of $p < 0.02$.

GeneSet	NGenes	Correlation	Direction	absLog2FC	*p* Value	Adj.*p*.Val
GO_REGULATION_OF_MESODERM_DEVELOPMENT	13	−0.0072	Down	0.29	2.16×10^{-03}	9.96×10^{-01}
GO_TRANSFERASE_ACTIVITY_TRANSFERRING_AMINO_ACYL_GROUPS	19	0.0022	Down	0.40	2.78×10^{-03}	9.96×10^{-01}
GO_ORGANIC_CATION_TRANSPORT	19	6.00×10^{-04}	Down	0.29	6.17×10^{-03}	9.96×10^{-01}
GO_OUTER_DYNEIN_ARM_ASSEMBLY	11	−0.0054	Down	0.28	7.87×10^{-03}	9.96×10^{-01}
GO_DENDRITIC_CELL_CHEMOTAXIS	12	0.0127	Up	0.19	8.46×10^{-03}	9.96×10^{-01}
GO_NEUTROPHIL_MEDIATED_IMMUNITY	22	0.0379	Down	0.27	8.90×10^{-03}	9.96×10^{-01}
GO_CYTOKINE_SECRETION	35	0.021	Up	0.34	1.16×10^{-02}	9.96×10^{-01}
GO_POSITIVE_REGULATION_OF_INNATE_IMMUNE_RESPONSE	231	0.0144	Up	0.22	1.16×10^{-02}	9.96×10^{-01}
GO_BETA_CATENIN_TCF_COMPLEX_ASSEMBLY	40	0.0151	Up	0.19	1.18×10^{-02}	9.96×10^{-01}
GO_AMMONIUM_TRANSMEMBRANE_TRANSPORTER_ACTIVITY	23	-1.00×10^{-04}	Down	0.18	1.34×10^{-02}	9.96×10^{-01}
GO_NEGATIVE_REGULATION_OF_STEM_CELL_DIFFERENTIATION	38	−0.0091	Down	0.32	1.38×10^{-02}	9.96×10^{-01}
GO_MHC_PROTEIN_BINDING	28	0.0209	Up	0.23	1.41×10^{-02}	9.96×10^{-01}
GO_ORGANIC_CATION_TRANSMEMBRANE_TRANSPORTER_ACTIVITY	15	0.0139	Down	0.25	1.63×10^{-02}	9.96×10^{-01}
GO_ACTIVATION_OF_INNATE_IMMUNE_RESPONSE	192	0.017	Up	0.21	1.67×10^{-02}	9.96×10^{-01}
GO_PROTEIN_AUTOPROCESSING	11	0.0355	Up	0.24	1.72×10^{-02}	9.96×10^{-01}
GO_ACTIVATION_OF_IMMUNE_RESPONSE	367	0.0158	Up	0.26	1.77×10^{-02}	9.96×10^{-01}
GO_REGULATION_OF_TYROSINE_PHOSPHORYLATION_OF_STAT5_PROTEIN	17	-1.00×10^{-04}	Down	0.24	1.82×10^{-02}	9.96×10^{-01}
GO_NOTCH_SIGNALING_PATHWAY	107	−0.0021	Down	0.31	1.85×10^{-02}	9.96×10^{-01}
GO_AXONEMAL_DYNEIN_COMPLEX_ASSEMBLY	20	0.0131	Down	0.27	1.99×10^{-02}	9.96×10^{-01}
GO_POLYSACCHARIDE_BINDING	20	0.0224	Down	0.58	2.00×10^{-02}	9.96×10^{-01}

Table S4. GO GeneSet assessment for the identification of pathways with differentially expressed genes in expanded osteoarthritic osteoblasts if cultured with AA, as compared to standard culture medium ($p < 0.01$).

GeneSet	NGenes	Correlation	Direction	absLog2FC	*p* Value	Adj.*p*.Val
GO_NEGATIVE_REGULATION_OF_DENDRITE_MORPHOGENESIS	15	0.008	Down	0.37	8.30×10^{-03}	9.95×10^{-01}
GO_OUTER_DYNEIN_ARM_ASSEMBLY	11	−0.0071	Down	0.24	1.07×10^{-03}	9.95×10^{-01}
GO_APOPTOTIC_SIGNALING_PATHWAY	282	-5.00×10^{-04}	Up	0.20	1.09×10^{-03}	9.95×10^{-01}
GO_3_5_CYCLIC_AMP_PHOSPHODIESTERASE_ACTIVITY	15	0.0151	Down	0.40	1.13×10^{-03}	9.95×10^{-01}
GO_VOLTAGE_GATED_CALCIUM_CHANNEL_COMPLEX	37	0.0098	Down	0.31	1.44×10^{-03}	9.95×10^{-01}
GO_RIBONUCLEOTIDE_CATABOLIC_PROCESS	28	−0.0119	Down	0.26	1.57×10^{-03}	9.95×10^{-01}
GO_AXONEMAL_DYNEIN_COMPLEX_ASSEMBLY	20	0.0147	Down	0.24	1.61×10^{-03}	9.95×10^{-01}
GO_EXTRINSIC_APOPTOTIC_SIGNALING_PATHWAY	96	−0.0013	Up	0.21	2.15×10^{-03}	9.95×10^{-01}
GO_EXTRINSIC_APOPTOTIC_SIGNALING_PATHWAY_VIA_DEATH_DOMAIN_RECEP TORS	38	1.00×10^{-04}	Up	0.16	2.34×10^{-03}	9.95×10^{-01}
GO_ZYMOGEN_ACTIVATION	103	-5.00×10^{-04}	Up	0.22	2.50E−03	9.95×10^{-01}
GO_NITRIC_OXIDE_METABOLIC_PROCESS	15	0.0107	Down	0.34	2.80E−03	9.95×10^{-01}
GO_POSITIVE_REGULATION_OF_PEPTIDASE_ACTIVITY	149	0.0016	Up	0.19	2.80E−03	9.95×10^{-01}
GO_CELL_DEATH	948	7.00×10^{-04}	Up	0.18	3.24E−03	9.95×10^{-01}
GO_ACTIVATION_OF_CYSTEINE_TYPE_ENDOPEPTIDASE_ACTIVITY	92	0.0014	Up	0.20	4.89×10^{-03}	9.95×10^{-01}
GO_REGULATION_OF_EXECUTION_PHASE_OF_APOPTOSIS	23	0.0025	Up	0.18	5.20×10^{-03}	9.95×10^{-01}
GO_CALCIUM_CHANNEL_COMPLEX	59	0	Down	0.25	5.25×10^{-03}	9.95×10^{-01}
GO_DEATH_RECEPTOR_ACTIVITY	24	0.0092	Up	0.32	5.39×10^{-03}	9.95×10^{-01}
GO_POSITIVE_REGULATION_OF_VASODILATION	29	0.0166	Down	0.46	5.45×10^{-03}	9.95×10^{-01}
GO_POLYSACCHARIDE_BINDING	20	0.0182	Down	0.46	5.85×10^{-03}	9.95×10^{-01}
GO_POSITIVE_REGULATION_OF_PROTEOLYSIS	356	0.0074	Up	0.18	5.95×10^{-03}	9.95×10^{-01}
GO_CYCLIC_NUCLEOTIDE_CATABOLIC_PROCESS	17	0.0294	Down	0.32	6.68×10^{-03}	9.95×10^{-01}
GO_SINGLE_ORGANISM_CELLULAR_LOCALIZATION	875	0.0033	Up	0.17	6.69×10^{-03}	9.95×10^{-01}
GO_MITOTIC_CELL_CYCLE_ARREST	13	−0.0372	Up	0.30	6.72×10^{-03}	9.95×10^{-01}
GO_RESPONSE_TO_ANGIOTENSIN	14	0.0033	Down	0.28	6.89×10^{-03}	9.95×10^{-01}
GO_PROTEIN_MONOUBIQUITINATION	51	0.0028	Up	0.13	7.58×10^{-03}	9.95×10^{-01}
GO_REGULATION_OF_CATABOLIC_PROCESS	717	0.0017	Up	0.16	7.87×10^{-03}	9.95×10^{-01}
GO_POSITIVE_REGULATION_OF_PROTEASOMAL_PROTEIN_CATABOLIC_PROCESS	98	0.0014	Up	0.15	8.06×10^{-03}	9.95×10^{-01}
GO_MACROMOLECULE_CATABOLIC_PROCESS	881	0.0064	Up	0.15	8.10×10^{-03}	9.95×10^{-01}
GO_INTERSPECIES_INTERACTION_BETWEEN_ORGANISMS	640	0.0171	Up	0.16	8.22×10^{-03}	9.95×10^{-01}
GO_REGULATION_OF_THYMOCYTE_APOPTOTIC_PROCESS	12	0.0239	Up	0.21	8.31×10^{-03}	9.95×10^{-01}
GO_ADRENERGIC_RECEPTOR_SIGNALING_PATHWAY	15	0.0105	Down	0.40	9.01×10^{-03}	9.95×10^{-01}
GO_REGULATION_OF_INTERLEUKIN_5_PRODUCTION	13	−0.0166	Down	0.38	9.11×10^{-03}	9.95×10^{-01}

GO_ADENYLATE_CYCLASE_ACTIVATING_G_PROTEIN_COUPLED_RECEPTOR_SIGNALING_PATHWAY	55	0.0226	Down	0.32	9.60×10^{-03}	9.95×10^{-01}
GO_CAMP_METABOLIC_PROCESS	32	0.0467	Down	0.36	9.60×10^{-03}	9.95×10^{-01}
GO_CELLULAR_MACROMOLECULE_LOCALIZATION	1205	0.002	Up	0.16	9.74×10^{-03}	9.95×10^{-01}

© 2017 by the authors. Submitted for possible open access publication under the terms and conditions of the Creative Commons Attribution (CC BY) license (http://creativecommons.org/licenses/by/4.0/).
